# Information Gradient among Nucleotide Sequences of Essential RNAs from an Evolutionary Perspective

**DOI:** 10.3390/ijms25147521

**Published:** 2024-07-09

**Authors:** Houssem Ben Khalfallah, Mariem Jelassi, Hajar Rissaoui, Mohtadi Barchouchi, Clément Baraille, Joël Gardes, Jacques Demongeot

**Affiliations:** 1 Laboratory AGEIS EA 7407, Team Tools for e-Gnosis Medical & Labcom CNRS/UGA/OrangeLabs Telecom4Health, Faculty of Medicine, University Grenoble Alpes (UGA), 38700 La Tronche, France; houssembenkhalfallah@gmail.com (H.B.K.); hajar.rissaoui@etu.univ-grenoble-alpes.fr (H.R.); mohtadi.barchouchi@etu.univ-grenoble-alpes.fr (M.B.); 2 ENSI—Ecole Nationale des Sciences de l’Informatique, Campus Universitaire de la Manouba, La Manouba 2010, Tunisia; 3 Orange Laboratorys, 38229 Meylan, France; clement.baraille@orange.com (C.B.); joel.gardes@orange.com (J.G.)

**Keywords:** origin of life, proto-ribosome, RNA ring, prebiotic systems, gradient of pentamer frequency

## Abstract

We hypothesize that the first ancestral “protocell” molecular structures, i.e., the first RNAs and peptides that gradually transformed into real cells once the Earth had cooled sufficiently for organic molecules to appear, have left traces in the RNAs and the genes in present cells. We propose a circular RNA that could have been one of these ancestral structures whose vestigial pentameric subsequences would mark the evolution from this key moment when the protocells began to join with living organisms. In particular, we propose that, in present RNAs (ribosomal or messenger), which play an important role in the metabolism of current cells, we look for traces of the proposed primitive structure in the form of pentamers (or longer fragments) that belong to their nucleotide sequence. The result obtained can be summarized in the existence of a gradient of occurrence of such pentamers, with a high frequency for the most vital functions (protein synthesis, nucleic synthesis, cell respiration, etc.). This gradient is also visible between organisms, from the oldest (Archaea) to the most recent (Eukaryotes) in the evolution of species.

## 1. Introduction

The oldest geological samples containing indisputable traces of life date back approximately 3.7 billion years [1]. We have no trace of previous lifeforms before the slow cooling of the Earth. To explain how life appeared, we propose a scenario by which events would have happened in two stages: (i) the acquisition of organic molecular structures that are typical of currently living cells and (ii) the acquisition of elementary functions that were necessary for the survival of the first living cells. These functions could have appeared in protocells, i.e., systems with two fundamental properties: (1) cooperation between the first peptides and ARNs, favoring the reproduction of both, and (2) a capacity to ensure redox transformations using basic substrates (methane, oxygen, hydrogen sulfide, etc.).

Following the discovery by Stanley Miller that small organic molecules (namely, nucleotides and amino acids) could appear spontaneously in the Earth’s atmosphere as it is assumed to have existed some 4 billion years ago [2], an intensive amount of research activity has been applied to determining the origin of life [3,4]. A huge body of literature has accumulated, with contributions ranging from biology to astrophysics (see, for instance, [5,6,7,8,9,10,11,12,13,14,15,16,17]). Two main streams of interpretative theories have followed, which are usually referred to as “DNA first” and “RNA first” [18]. 

However, although a century has passed since the pioneering article by Alexander Oparin [19], the ambitious project to develop a mixture of reagents that are capable of changing into a living system [20,21,22] is far from being achieved. However, in light of the discovery of the existence of a nucleic oligomer called AL (for Archetypal Loop [23,24,25]), which could have marked the transition process from protocells to real cells, we propose that, in the nucleotide sequences of RNAs (ribosomal or messenger), which play an important role in the metabolism of current cells, we can look for traces of this primitive structure in the form of pentamers (or longer fragments) that belong to their nucleotide sequence. The result obtained can be summarized in the existence of a gradient of occurrence frequency of such pentamers, with a high frequency for the most vital functions (protein synthesis, nucleic synthesis, cell respiration, etc.). This gradient is also visible between organisms, from the oldest (Archaea) to the most recent (Eukaryotes), in the evolution of species.

In the first section, we recall the properties of the archetypal AL structure; then, in the second section, we systematically search for traces of pentamers that originate from the nucleotide sequence of AL in RNAs (rRNAs and mRNAs) of organisms, ranging from the most ancient (Archaea) to the most recent (Eukaryotes). We demonstrate the existence of a frequency gradient among these pentamers and, finally, in the third section (entitled Discussion), we demonstrate the coherence of our approach using phylogenetic results from the literature and those from a new classification, based on the use of the Maxwell© classifier [25].

## 2. Results

### 2.1. First Works on the Origin of Life

No form of life, including microbial, appeared in an adequate nutrient medium in the absence of any source of contamination, according to Louis Pasteur [26,27] and others. It was not possible for these researchers to perform an infinite number of experiments, and a rare event could very well have escaped them. Therefore, these experiments do not prove that the probability of a “non-living → living” transition is strictly zero, but only that it is very low. However, living beings, including those that are the least evolved, are the seat of complex cross-regulated processes. How could inert matter change directly, as if by the wave of a magic wand, into such a finely regulated system? In short, the rule that “*a living being can only arise from an already living being, while the “non-living → living” transition is impossible*” appears to be inescapable. That said, this was the case at Pasteur’s time, i.e., at a time when the Earth had become relatively calm, but it leaves open the possibility that something crucial could have happened on the nascent Earth.

The solar system, including our Earth, arose from the gravitational collapse of a gigantic cosmic cloud of gas and dust approximately 4.568 billion years ago. This has been taken as instant zero in the age of the solar system. What followed was a complicated series of violent events over 1 billion years, followed by a cooling period that was more favorable to the appearance of life [28]. 

### 2.2. Protocell Structures

Protocell structure would have started, during this cooling period, to involve organic molecules, which were at first simple, and then became more and more complex. After up to, perhaps, several hundred million years of this functioning, one or more of these protocells would have acquired the organic equipment and the structure of real cells. The steps considered in the classical theories (DNA first or RNA first) are suitable for explaining how this would have happened, and we have proposed the AL RNA ring [23,24,25] as an example of a molecular structure that marks the transition from purely mineral behavior to the involvement of organic molecules, by using genomic sequences from multiple species from [29,30,31,32].

It is possible that the organization of primitive functional living molecular structures took place inside a primitive cell. The hypothesis of prior evolution in a conglomerate-type structure of RNA subunits and peptides, as in the current ribosome, is also possible. This would not require the existence of a proto-membrane and could give rise to the appearance of random peptides whose biosynthesis would be favored by the presence of circular RNA-type catalysts, for example, the RNA AL ring [32]. After the appearance of proto-membrane structures made up of lipids and peptides, isolating an interior environment made up of a proto-ribosome, primitive functional living molecular structures could continue to evolve, so as to provide increasingly complex functions, giving a selective advantage to pre-cells that possess them. This would explain the existence of a proximity gradient, in terms of common fragments, to primitive peptide-catalyzing RNAs, such as the RNA AL ring.

### 2.3. The AL RNA Ring as an Ancestor of the Ribosome?

**Figure 1:**
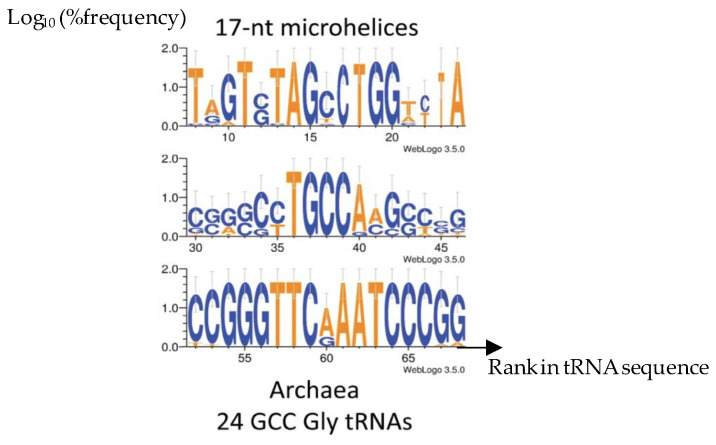
Consensus content of proto-tRNA minihelices of tRNA-Gly^GCC^ from 24 Archaea in [38].

Considering the primary structure of the tRNA loops of Archaea, the same pentameric sequences occurred first in many species, like those belonging to the ancient Archaea studied in Figure 1, showing the following motifs: TGGTA for the D-loop, CTGCCA for the Anti-codon loop, and TTCAA for the Tψ-loop [33,34,35,36,37]. Examples of such small sequences are given in Figure 1 and Table 1.

**Figure 2:**
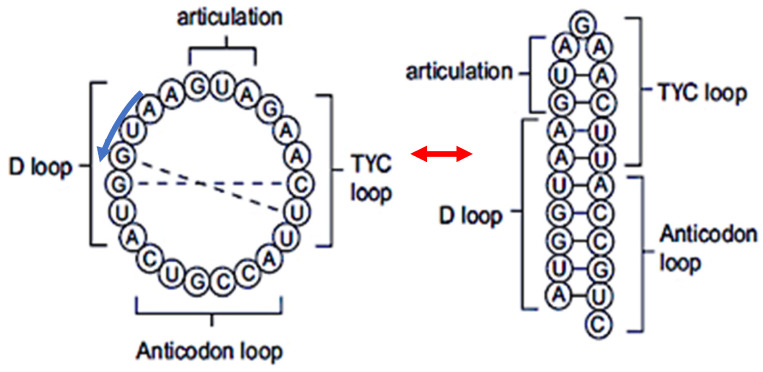
(**Left**) ring form of AL sequence. (**Right**) hairpin form of AL sequence. The two forms are in equilibrium (red arrows) and are read in the 5′-3′ sense (blue arrow).

From Figure 1 and Table 2, it is easy to find that the following 19-nucleotide-length consensus sequence, defined from tRNA-Gly loops: 5′-AAUGGUACUGCCAUUCAAG-3′, has a secondary hairpin form if we add AUG (as start codon) on the 5′ extremity. This hairpin form is described in Figure 2 (right) and its ring form in Figure 2 (left). This sequence that we call AL (for Archetypal Loop or ALpha ring) has some optimal combinatorial properties, which suggest that it could have played a catalytic role in primitive peptide genesis [33,34].

The choice of the tRNA-GlyGCC comes from the fact that its anticodon loop is the most frequent in archaeal tRNA-Gly [39] (see the 11 tRNA-GlyGCC in Table 2 and 246 more in the Supplementary Material in [29,30]). The combinatorial properties result from opposite constraints in a min–max optimization problem and can be summarized as follows [34]:

(i). If the ring form has to play a reactive role with respect to the amino acids in order to facilitate their polymerization (as a “marriage agency”), taking advantage of the affinities between amino acids and the codons and anticodons of their specific synonymy classes in the genetic code [40], the ring has to be short. If the ring has to survive in a stable hairpin form with minimal free energy, it presents a self-complementarity between its two half-parts;
(ii). Among the rings that satisfy the principle “to be as short as possible and containing at least one codon of each amino-acid”, there is no solution for a length below 22 nucleotides. For the length of 22, 29,520 among 1,761,011 solutions contain only 1 repeated codon AUN, with N being G for 52% of the solutions;
(iii). From these 25 rings, 19 encompass both the start and stop codon UGA;
(iv). Through the calculation of several distances (e.g., circular Hamming distance, permutation distance, and edit distance), the ring AL (Figure 2, (left)) exhibits a minimum average distance as compared to the others. Thus, only this sequence acts as the barycenter of the set of the 18 others.

From detailed studies [35,36,37], it appears that pentamers or hexamers from AL could have been remnants on the way to the progressive construction of nucleic acids that were involved in ribosome building and in the mRNA of enzymes that are involved in ATP metabolism. Thus, following the concentration of these small sequences in many primitive species (Archaea or Bacteria) during the evolution makes it possible to obtain information on what could have happened during a long period for which we do not have fossils or any other sources of data. Study is continuing now towards the intervention of these small RNAs in the progressive construction of the ancestors of the current ribosome [41,42].

**Figure 3:**
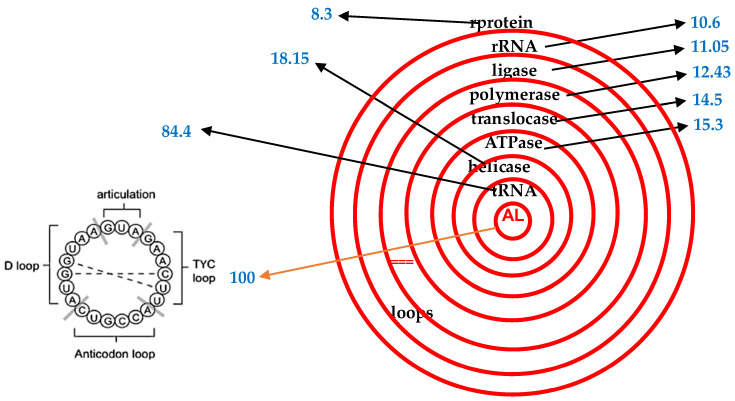
(**Right**) Mean AL pentamer proximity (in blue) for the four species in Table 1 [29,30]. (**Left**) AL ring.

This is carried out by using the proximity, denoted PpAL (for Pentamer proximity to AL), of a given sequence of nucleotides, equal to twice the number pAL of standard deviations in the difference between the observed and expected numbers of pentamers (chosen from a set of 9 pentamers: ATTCA, TTCAA, TCAAG, CAAGA, AAGAT, AGATG, GATGA, ATGAA, TGAAT located at the head of the hairpin form of AL and easy to fragment from the rest of the hairpin) that are common between AL and this sequence. PpAL supports some current quantitative phylogenies that have been proposed in the absence of any identified root. This root could be an AL ring, as a possible LUCARN (Last Universal Common Ancestor RNA). In Table 2 and Figure 3, we give the PpAL for functions and molecules of four species; PpAL ranking respects both the gradient of seniority (for species) and necessity (for functions).

To be more precise, we have chosen the seven pentamers at the head of the hairpin form of AL, of which at least one nucleotide shares only two bonds with its neighbors, plus the two adjacent pentamers, making the hypothesis that these fragments are more unstable than those in the body of the AL hairpin configuration. If we find these nine pentamers in an RNA with an observed frequency that is much higher than the expected frequency (equal to 9/1024), then we are talking about traces (or relics) of the AL sequence in the sequences studied. If this observed frequency is greater than 4 standard deviations from the expected frequency (which is generally the case for the sequences studied), then, in the Gaussian approximation of the binomial law, the probability of such an event is less than 5·10^−5^, which justifies the use of this method to quantify the “proximity” of a given sequence to the AL “reference”.

### 2.4. A Potential Role of AL Protocells to Join the Organic World

The AL structure, which has two conformations (ring and hairpin) and is a possible vestige on the way to the construction of ribosomes, already provides solid data on this period without fossils, and we may hope to obtain more in searching for new nucleic oligomers through the study of other nucleic acids and/or other living species [34,35,36,37].

One of the major problems in evolution after the appearance of autocatalytic molecular systems, as centered on a primitive nucleic oligomer ancestor of the tRNAs and ribosomal RNAs [38,39], is related to the appearance of the cell membrane, which isolates these primitive systems from the outside world and promotes their survival. The small peptides resulting from the interaction between the RNA ring AL and amino acids can give rise to the diffusion of the polymerizing peptide material in the vicinity of the initial nucleic structure. We can imagine that such structures segment the space by prefiguring cellular proto-membranes [40]. Similarly, the interaction between nucleo-peptide structures and lipids synthesized in the primitive atmosphere can lead to a space-separating proteo-lipidic organization by reinforcing the initial peptide proto-membranes, as in the current mode of trans-membrane peptides that are fixed in phospho-lipid membrane pores or by acting as rafts in a lipid medium locally organized like a liquid crystal [41,42,43,44,45,46,47,48,49,50].

## 3. Discussion

**Figure 4:**
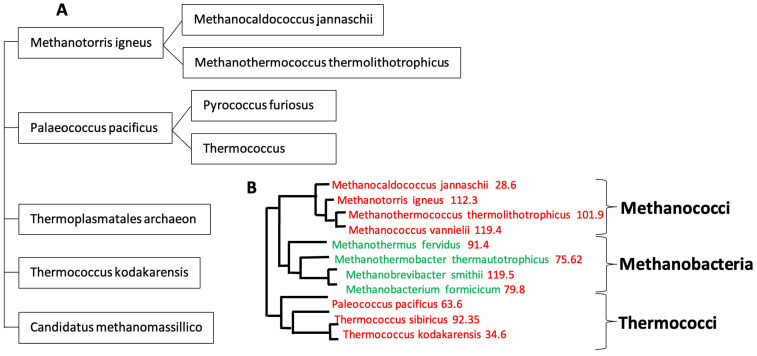
(**A**) Maxwell© clustering of Archaea. (**B**) p_AL_ proximity (in red) of Methanomada and Thermococci on the Archaea phylogenetic tree, based on ribosomal proteins (L7–L12, L30, S4) [52].

**Figure 5:**
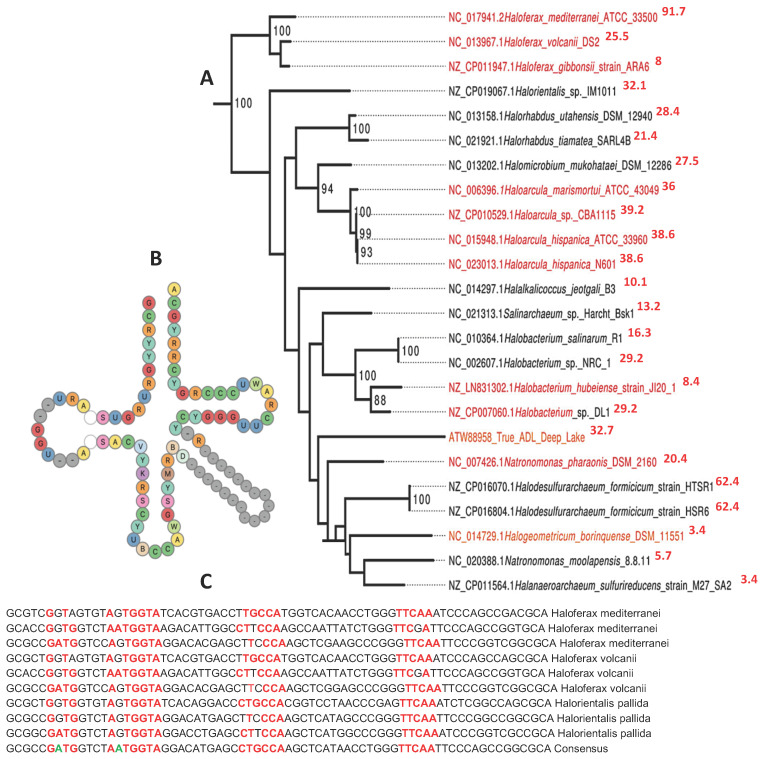
Haloferacaceae (**A**): phylogenetic tree obtained using Methyltransferase mRNA sequences (based on [53]) with indication (in red) of a proximity to AL based on 5 pentamers; (**B**): consensus secondary structure tRNA-Gly [33]; (**C**): consensus primary sequence tRNA-Gly [29,30].

In [51], we have used a new classifier reversible (able to make explicit and give explanation on its reasoning during all the steps of clustering) and agnostic (not using a priori semantic knowledge on the data) called Maxwell©. In Figure 4, we see the Pp_AL_ for the whole genomes of Methanomada Archaea clusterized by the classifier Maxwell© [51] and a phylogenetic tree [52], with indication of the Pp_AL_ (in red) respecting the phylogeny ranking. In Figure 5, we observe the same phenomenon for the PpAL of the whole genomes of halophilic Archaea, which gives consistency to the method of classification based on the Maxwell© classifier and also gives pertinence to the use of proximity to AL based on only 5 pentamers (AGATG, GATGA, GTGGC, TGGCC, GGCCT from the extremities of 1 of the 18 hairpins, respecting the conditions (i) to (iii) of AL construction), which roughly respects the biological phylogeny previously obtained from the Methyltransferase mRNA sequence clustering of the concerned Archaea species [53].

**Figure 6:**
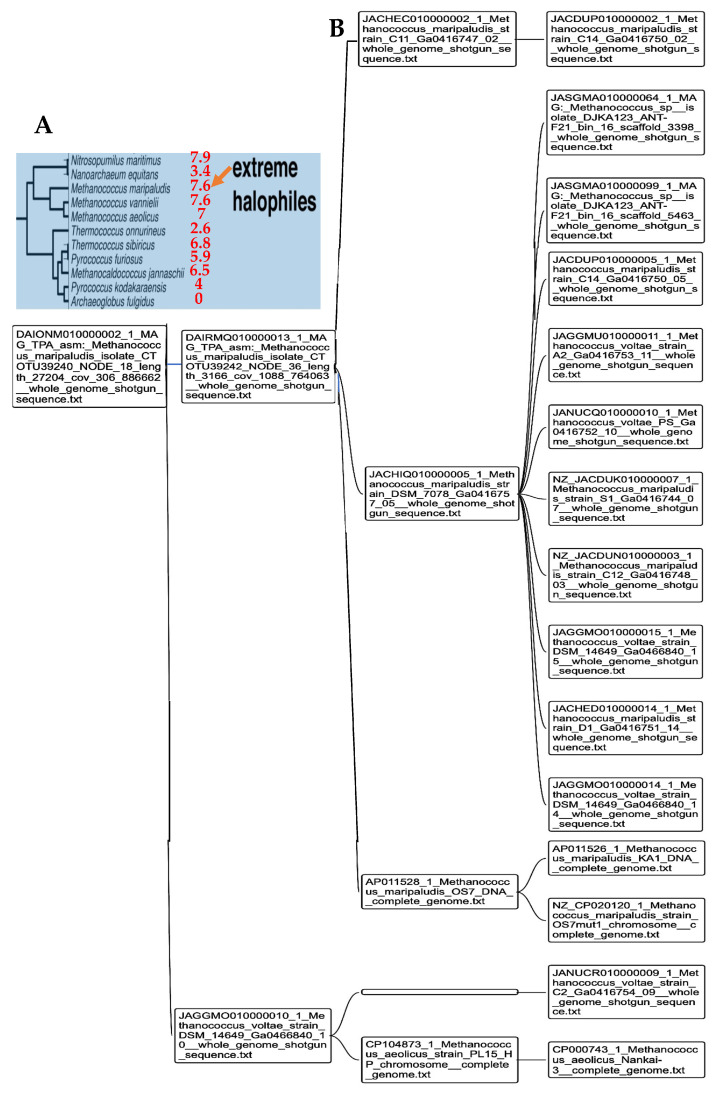
(**A**) Indication of p_AL_ (in red) for extreme halophilic Archaea on a phylogenetic tree obtained using a non-redundant set of proteomic features [56]; (**B**) Methanococcus phylogeny obtained using the Maxwell© classifier and based on the whole genome.

Figure 6 shows the AL pentamer proximity of the whole genome of extreme halophilic Archaea belonging to the Methanococcus genus (from Methanococcaceae family [54]), and this proximity is in agreement with the clustering obtained using the Maxwell© classifier. Only Thermococcus sibiricus presents a negative proximity, due probably to the fact that it is the target of a virus whose genetic sequence has contaminated the whole genome of Thermococcus sibiricus [55,56].

The adequacy between the results of a classification based on a choice of the mRNAs of ribosomal proteins, an agnostic classification based on the entire genome, and calculations of proximity to AL, showing relics of its fragments in the genome of current species in connection with their seniority, reinforces the idea that AL structure could have participated as primordial RNA in the mechanisms involved at the origin of life.

In this spirit, we have proposed, in previous articles [33,34,35,36], that the AL sequence found, for example, in the succession of loops of numerous tRNAs that belong to numerous species (in particular, Archaea) could have behaved as a primitive catalyst for random peptides. The combinatorial properties of the circular configuration of the AL sequence are remarkable, as they provides, with minimal length, weak binding sites for all amino acids. The AL configuration can indeed be either a ring or a hairpin, depending on the environmental conditions. The form ring is functional because it allows, on a sequence of minimum length, to provide all amino acids with weak binding sites at their codons [57,58], which can, therefore, by proximity create more stable peptide bonds. However, these codons appear, for each class of synonymy of the genetic code, once and only once by overlap in a ring-type structure; therefore, they are optimal for this function of the “marriage agency” of amino acids. The hairpin structure allows the conservation of the sequence outside of its catalytic function.

The aim of this article is to show that, in addition to having left traces in tRNAs and rRNAs [50,51], it has also impacted the mRNAs of numerous proteins that are essential for the survival of the species which synthesize them—these proteins being hypothetically derived from peptides randomly manufactured by the primitive AL ring machinery. It is this hypothesis that we wanted to test. The result is not definitively conclusive, but invites us to continue mapping proteins and species, using the marker of proximity to AL and trying to find a logic to the observed correlations (correlations with the antiquity of species and with the critical aspect for the survival of the chosen proteins). This work will be very long, but it must be carried out to affirm the existence of traces of a functional advantage transmitted from the primitive genome to evolved genomes.

**Figure 7:**
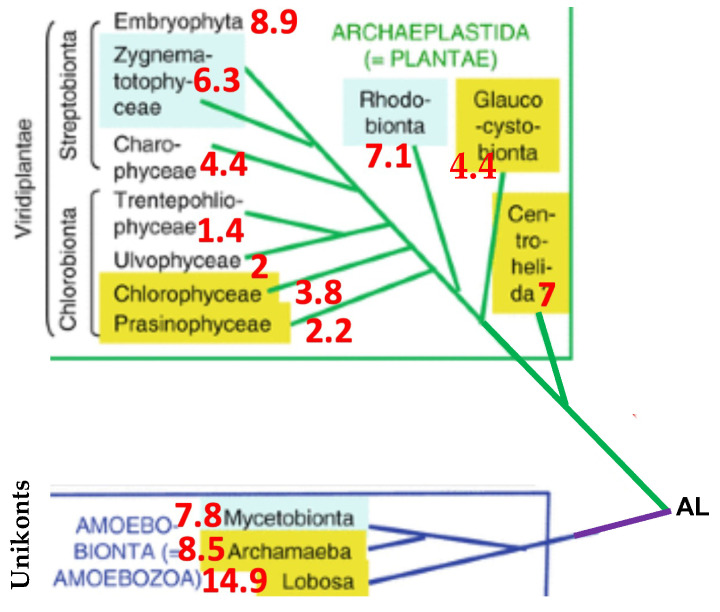
Phylogeny of some unicellular eukaryotes (from [58]), with the indication of pAL (in red).

In this spirit, let us consider, for example, in Figure 7 the phylogeny of certain ancient algae [59,60]: Archaeplastida is a major taxonomic group of eukaryotes, comprising the photoautotrophic red algae (Rhodophyta), green algae, land plants, and the minor group glaucophytes, with no centriole and with mitochondria having flat cristae. and Amoebozoa is a taxonomic group containing amoeboid protists, often possessing lobose pseudopods, blunt, fingerlike, and tubular mitochondrial cristae. We see, in Figure 7, that the hypothesis of the existence of a correlation between the presence of fragments from the RNA AL ring within crucial proteins, such as helicase, and the antiquity of species containing these proteins (attested by classical phylogenies) is not systematic, but the trend exists and must be confirmed for Eukaryotes in subsequent studies concerning more species and proteins that are more functionally important for their survival (see also Supplementary Material for the localization of AL pentamers in the species in Figure 7).

The unicellular eukaryotes represent about 10 percent of all eukaryotic species, and they show a huge phyletic diversity. The chosen species have in common their chloroplast structure, photosynthetic pigments, cell wall, and kinetic apparatus, and they belong to two monophyletic ensembles: Archaeplastida (called also Plantae) and Amoebobionta (called also Amoebozoa).

## 4. Materials and Methods

We use the following different public databases (Table 3) for identifying the most frequent sequences of the tRNA loops in different realms of life, as well as for calculating the free energy of some hairpin structures and for counting the occurrence of pentamer motifs in gene sequences.

## 5. Conclusions

In the protocell scenario, there is no straightforward transition from an inert mixture of chemical substances to a living cell. Life on Earth could have appeared as a progressive acquisition of the molecular characteristics of real cells from the moment the Earth had cooled sufficiently for organic molecules to exist. There is no evidence that protocells really existed; however, structures such as the RNA ring AL came at the right time to mark the crucial moment when the processes involved would have reached the frontier between the organic world and life realm: RNA rings could have co-existed in equilibrium with DNA hairpins that had the same nucleotide sequence inside lipidic vesicles that also contained amino acids and sugars [61,62]. The protocell scenario is far from answering all the questions that one can ask about life and its origin, but it at least underlines the need to take into account the progressive acquisition of some characteristics of life and to propose a logical way for this to have occurred.

We propose, as a follow-up to this work, to systematically explore many other species in the kingdoms of archaea, bacteria, and eukaryotes to confirm whether the vital functions examined in this article and others that have been presumed less important for survival present the same gradient of proximity to the RNA ring AL, according to their supposed ancient (for species) and necessary (for functions) character.

## Figures and Tables

**Table 1:** Some species whose tRNA-Gly^GCC^ primary sequence have the same constant motifs (in red) in their loops [29,30].

Species	Articulation D-Loop Anticodon-Loop Tψ-Loop
*Methanococcus maripaludis*	GCGGCTTTGATGTAG ACTGGTATCATACGGCCCTGCCACGGCCGACACCCGGGTTCAAATCCCGGAGGCCGCA
*Methanococcus vannielii*	GCGGCTTTGATGTAG ACTGGTATCATACGGCCCTGCCACGGCCGACACCCGGGTTCAAATCCCGGAGGCCGCA
*Methanococcus voltae*	GCGGCCTTGATGTAG TGGTATCATACGGCCCTGCCACGGCCGATACCCGGGTTCAAATCCCGGAGGCCGCA
*Methanocaldococcus jannaschii*	GCGGCCTTGGTGTAG CCTGGTAACACACGGGCCTGCCACGCCCGGACCCCGGGTTCAAATCCCGGAGGCCGCA
*Halorhabdus utahensis*	GCGACGGTGGTGTAGTGGTATCACAGGACCCTGCCACGGTCCTAACCCGAGTTCAAATCTCGGCCGTCGCA
*Petromyzon marinus* (lamprey)	GCATCGGTGGTTCAGTGGTAGAAATCTCGCCTGCCACGCGGGAGGCCCGGGTTCAATTCCCGGCCGATGCA
*Danio rerio* (zebrafish)	ACATTGGTGGTTCAGTGGTAGATTTCTCGCCTGCCACGTGGGAGGCCCGGGTTCAATTCCCGGCCAATGCA
*Strongylocentrotus purpuratus*	GCATTGGTGGTTCAGTGGTAGAATTCTCGCCTGCCACGCGGGGGACCCGGGTTCAATTCCCGGCCAATGCA
*Loxodonta africana* (elephant)	GCATTGGTGGTTCAGTGGTAGAATTCTCGCCTGCCACGTGGGAGGCCTGGGTTCAATTCCCAGCCAGTTCT
*Callithrix jacchus* (marmoset)	GCATGGGTGGTTCAGTGGTAGAATTCTCGCCTGCCACGCGGGAGTCCTGGGTTCAATCCCCGGCCCACGCA
*Arabidopsis thaliana*	GCACCAGTGGTCTAGTGGTAGAATAGTACCCTGCCACGGTACAGACCCGGGTTCAATTCCCGGCTGGTGCA
*Medicago truncatula*	GCACCAGTGGTCTAGTGGTAGAATAGTACCCTGCCACGCTACAGACCCGGGTTCAATTCCTGGCTGGTGCA

**Table 2:** AL pentamer proximity (Pp_AL_) for Homo sapiens (HS), Saccharomyces cerevisiae (SC), Methanococcus voltae (Mv), and isolate (Mmi).

Molecule	Species	Pp_AL_ = 2P_AL_/σ	n_o_	N	n_e_	(σ_e_)	p_AL_	Mean Pp_AL_
**rprotein L18**	HS	**7.4**	**13**	552	4.9	(2.2)	**3.7σ**	**8.3**
	SC	**10**	**16**	557	4.9	(2.2)	**5σ**	
	Mv	**6**	**12**	581	5.1	(2.26)	**3σ**	
	Mmi	**9.8**	**16**	578	5	(2.25)	**4.9σ**	
**rRNA 5S**	HS	**2**	**2**	117	1	(1)	**1σ**	**10.6**
	SC	**2**	**2**	117	1	(1)	**1σ**	
	Mv	**26.2**	**14**	112	0.9	(1)	**13.1σ**	
	Mmi	**12.2**	**7**	111	0.98	(0.99)	**6.1σ**	
**Gly-tRNA ligase**	HS	**8.8**	**36**	2015	17.7	(4.2)	**4.4σ**	**11.05**
	SC	**13**	**45**	2000	17.6	(4.2)	**6.5σ**	
	Mv	**11**	**37**	1751	15.4	(3.9)	**5.5σ**	
	Mmi	**11.4**	**37**	1721	15.1	(3.9)	**5.7σ**	
**DNA polymerase**	HS	**5.8**	**21**	1286	11.3	(3.36)	**2.9σ**	**12.43**
	SC	**9.5**	**36**	1895	16.6	(4)	**4.75σ**	
	Mv	**16.2**	**59**	2472	21.7	(4.6)	**8.1σ**	
	Mmi	**18.2**	**30**	749	6.6	(2.6)	**9.1σ**	
**Translocase**	HS	**5.4**	**42**	3178	27.9	(5.3)	**2.7σ**	**14.5**
	SC	**26.8**	**130**	4856	42.7	(6.53)	**13.4σ**	
	Mv	**19.6**	**76**	2969	26	(5.1)	**9.8σ**	
	Mmi	**6**	**22**	1325	11.6	(3.4)	**3σ**	
**ATPase**	HS	**7**	**29**	1755	15.4	(3.9)	**3.5σ**	**15.3**
	SC	**12.8**	**42**	1850	16.26	(4)	**6.4σ**	
	Mv	**13.6**	**21**	608	5.34	(2.3)	**6.8σ**	
	Mmi	**27.8**	**97**	2978	26.2	(5.1)	**13.9σ**	
**Helicase**	HS	**12.6**	**48**	2255	19.8	(4.45)	**6.3σ**	**18.15**
	SC	**26.6**	**95**	3029	26.6	(5.16)	**1** **3.3** **σ**	
	Mv	**15.2**	**57**	2465	21.7	(4.65)	**7.6σ**	
	Mmi	**18.2**	**60**	2236	19.6	(4.4)	**9.1σ**	
**tRNA-Gly**	HS	**81**	**18 **	22	0.19	(0.44)	**40.5σ**	**84.4**
	SC	**76.4**	**17 **	22	0.19	(0.44)	**38.2σ**	
	Mv	**85.5**	**19 **	22	0.19	(0.44)	**42.8σ**	
	Mmi	**94.6**	**21**	22	0.19	(0.44)	**47.3σ**	
**AL**		**100**	**22**	22	0.19	(0.44)	**50σ**	**100**

**Table 3:** Websites for data sources, accessed on 15 April 2023 [29,30,31,32].

tRNA database	http://lowelab.ucsc.edu/GtRNAdb/Lafri3/Lafri3-align.html, accessed on 23 March 2024 http://trna.bioinf.uni-leipzig.de/DataOutput/Result, accessed on 23 March 2024
Secondary structure	http://trna.ucsc.edu/tRNAviz/, accessed on 23 March 2024
Gene sequence	https://www.ncbi.nlm.nih.gov/nucleotide?cmd=search, accessed on 23 March 2024

## Data Availability

All the data are obtained from readily available public databases that are cited in the text.

## Supplementary Materials

The following supporting information can be downloaded at: https://www.mdpi.com/article/10.3390/ijms25147521/s1.
