# Supplementary material for: Information Gradient among Nucleotide Sequences of Essential RNAs from an Evolutionary Perspective
Source: Int J Mol Sci. 2024 Jul 9;25(14):7521. doi: 10.3390/ijms25147521 (PMC11277137; doi:10.3390/ijms25147521)
Supplement: Supplementary file 1 [file ijms-25-07521-s001.zip › ijms-3040906-supplementary.pdf]

Table S1. mRNA sequences of helicase (with T instead of U) for various species from two taxonomic groups of eukaryotes, Archaeplastida and Amoebozoa, with indication (in various colors) of AL-pentamers and calculation of the AL pentameric proximity.

>Corynoplastis japonica strain NIES-2662 chloroplast sequence GenBank: KY709210.1: 154864-154911,158598-160265 helicase glaucocystobionta

mRNA Sequence

ATACCACCTTATAATTTATTAGCAGAAGAAATAATAGTTGGAGGTATCTTATTAGATCCAAAAAT
TATTGTAAACATAGCACATAAATTAAAACCAGAAGCTTTTTACTTTTTACCTCATAAGCTAATTT
ATGAAGCTATATTTAATCTTTACATGAGTGGTCATCCAGTTGATTTGTTATCTATAATTTATAGT
TTAAGAAGTCAAGAAATTTTAGTTAAAGTTGGAGGAATTGAAAAAATTCTTTATTTTATGGATC
AAACATTTACTAATTTACATTTTGATGATTATGTCGCTTTAATTGTAGATAAATATATTAGAAG
ATCATTAATACAATATGGCTCAAATTTAATTAATTCTAGTTATAGTATTGTTCCTATAGATACAA
TTATCTCAACAGTTGAAAAAGATATGGCTGATATAACTCAAAGTTATCACTATCAAGATTTATAT
TCTATGTCTAAATTGCTTAAGACTACTTTATCTAATATTTCAAATCCTTCTTTAATAAATCAAGT
TGCTGGAATACCATCAGGGTTTTATGGTCTGGATGCTATTACTCAAGGTTTTCAGAATTCAGATT
TGATTATCATAGCAGGTAGACCCTCTATGGGTAAAACTGCATTTTGTTTGAATATAGCCTACAAC
ATTACCAATGCTATTCATATACCTATAGCAATTTTTAGTTTAGAGATGACAAAACAACAATTAAT
TTATAGACTGTTATCAATATCTTCGTTAATACCTAGTAATCGTTTACAGTCCGGTAGAATCAATT
ATGATGATTGGTCTAAATTATATGATTCGATATCTTCACTATCTCGTTTTAAAGTCTATTTAGAT
GACACTCCGAATTTATCTACTACAACTATTAGAACTAAAACAAAAAAATTAAATCAACAAGAAA
AAAATTTAGGATTAATTATAATTGATTATTTACAACTTATTCAGAGTTCAATAGGAGAAGATAG
TAGAAGTCAAGAGTTGTCAATTATTACTAGATCATTAAAATCTCTAGCACGTGAGCTTAATTTAC
CAGTTATAGTATTGTCACAATTAAGTCGTAATGTTGAAGTACGAAATAATAAAAAACCATTGCT
ATCAGATTTGAGAGAAAGTGGTTGTCTTACAGGTAATACTTTAATTAATTGTATAAAAAATGAG
TTAATAGCTATTAAACAATTTAATCGTTACTTGCATCGTTATATTTTAAATTTGAACTTTAGTAG
TTTTAAGTTAATATATTCAGATCAATTAAAGTTCATGTATCATGGGTTTAAATATGTATATTTTT
TATCTATAGCACGGAATAAAATTATTAATTTAACAGCTAATCATAAAGTTTTAACTAAACGTGGT
TGGATACGAATTGATAATTTATATATTATTGATCAAATAGCGACCCTTAGTTGTCCTGATGATAT
CAACTTTAATTTTGTACCGCTAGTAAGTATTAATTTATTTAGTAAACAATTAACATATGATTTAG
GGGTCAAACCATTTAAAAATTTCCTTGCCAATACTGTTATTGTCCATAATTCTATTGAACAAGAT
GCAGATTTAGTGTGTATGCTCTATCGTGAAGATTATTATAACCCTCAAACCGAGTATGCTAATAT
CGCAGAAATTATTGTTGCTAAACATAGGAATGGCCCAACAGGAAGCTTCGAGTTAATTTTTGACC
CTTACACTACAACTTTTAAAAATATAGAGTAA

Occurrences/Results 9 Pentamers

The pentamer 'ATTCA' occurred 4 times.
The pentamer 'TTCAA' occurred 2 times.
The pentamer 'TCAAG' occurred 5 times.
The pentamer 'CAAGA' occurred 5 times.
The pentamer 'AAGAT' occurred 6 times.
The pentamer 'AGATG' occurred 3 times.
The pentamer 'GATGA' occurred 5 times.
The pentamer 'ATGAA' occurred 1 times.
The pentamer 'TGAAT' occurred 1 times.
The total occurrences of all specified strings is 32.
The total number of characters in the text is 1712.
First result (Total characters *9 /1024): 15.08203125
Square root of the first result: 3.883559095726496
Final result: 4.356305217195404

>Shewanella algae TUM17386 DNA, complete genome GenBank: AP024617.1: 896153-900028 helicase

mRNA Sequence

TTGAACGCTAAGCTGCACCCACTCTCCCGCGAATATCTGGATCTCTGCTTTCAGGCCGATGCGGCG
CGCATTCGCCGCAGCCTGTTCCGCTTGAAAAAAAGCCCCGATACAGAAGCCAAACAGCAAAAACT
CGCCGAGCTCAGTGAAGCTGCGGTACAGGCTTATGAAAGGGCGCAGCAAAGGCTGGCAGACAGAC
CTGTCATCGAGTATCCGGATGAGCTGCCGGTGTCACAAAAGCGCGATGAAATCGCCTCGGCCATC
ATCAATCATCAGGTGGTGATAGTGGCCGGTGAGACCGGCTCGGGCAAGACCACTCAGCTGCCCAA
AATCTGCCTCGACTTGGGGCTTGGCAGCCGTGGTCTGATTGGCCACACTCAGCCCAGGCGGCTGGC
GGCCCGCAGTGTGGCCAGCCGGGTTGCCGATGAACTCAAGACAGAGCTGGGTCAGGCGGTGGGTT
TTAAGGTTCGTTTCGCCGATGCGGTTTCCCAGGACTCCTATATCAAGTTGATGACAGACGGTATC
CTGCTGGCAGAACTGACATCGGATAAATGGCTCAGTCAGTATGATTGCCTCATCATAGACGAGGC
CCATGAGCGCAGTCTCAACATCGACTTTATTCTGGGTTATCTGAAGCAAATTTTGCAAAAGCGCC
CGGATCTCAAGTTGATCATCACCTCGGCCACCATAGATGTGGAGCGTTTTTCCAGGCATTTTGAC
AATGCGCCCGTGGTGGAAGTGTCCGGCCGCACCTATCCGGTGGAAACCCGCTATCGCCCCCTGTTG
CAGGATGACGACGCCGATTTGGATCTGATGGACGGCATCTTTGCCGCCGTCGATGAACTCTGTCG
TGAAGGCCCGGGGGACATTCTGATTTTCATGAATGGCGAGCGCGAAATCCGCGATACCGCTGAGC
AGCTGAAAAAGCGTAACCTGCGCGATACCGAGATCTTGCCGCTTTATGCCCGTTTGTCATACGGC
GAGCAATCCAAGGTATTCAAGCCGCATCCAGGGCGGCGCATTGTATTGGCCACCAACGTGGCCGA
AACCTCGCTGACTGTGCCCGGGATCCGCTATGTTATCGACCCAGGTACCGCCAGAATAAGTCGCTA
CAGCTATCGTACCAAGGTGCAGCGTTTGCCGATTGAGCCCATCTCCCAGGCCAGTGCCAATCAACG
CCAGGGACGTTGCGGCCGGGTTGGACCGGGGATCTGTATTCGCCTCTATGACGAACAGGACTTTA
TCAATCGGCCGGAGTTTACCGATCCTGAAATTCTGCGCACCAACCTGGCATCCGTGATCCTGCAGA

TGTTGGCCATAGGCCTGGGTGACATTGAGGGCTTCCCCTTCATTCAGCCACCGGATCCGCGCCATA
TCCGCGACGGCTTCCTGCTGCTCGAAGAGTTGCAGGCGGTGAGCAAGCACAAAGGGGCGCTGAAA
CTGACCCCGCTCGGGCGCGATCTGGCGAAGATCCCGGTCGATCCCCGCTTGGCCCGCATGGTGCTT
GAGGCCAACAAGCTGGGTTGTCTGCATGAGGTGATGGTGATTGTCGCCGGGTTATCGATTCAGGA
TCCCCGCGAGCGGCCACATGAGAAGAAACAGGCCGCCGATGAGGCCCATCGCCGTTTTGCCGATA
AAGACTCCGACTTTGTTGCCTGGCTAAATCTGTGGCAGTACCTCAAAGAGCAACAGAAGAGTCTG
TCATCCAGCCAGTTCCGCAAGCAGGTGAAGGCCGAGTATCTGGCCTATTTGCGTATTCGCGAATG
GCAGGATCTCTATGCCCAGCTGCGTCAGAGTGTGCACGACCTCAAGTGGCGCCTCAATAGTCAGG
CCGCCGAATATGAGCCACTGCACCAGGCGCTGCTCTCGGGGCTGCTGAGCCATATCGGTTTCAAGG
ATAAAGACAACGAGTATCTGGGGGCCCGCAACCGGCGCTTCTTTGTGTTTCCCGGCTCGCCGCTGG
CCAAGAAGGGCCCCAAGTGGATTATGGCGGCCGAGCTTACCGAAACATCACGCCTGTTCGCCCGT
ACCTGCGCCCGTATAGACCCTGTATGGCTCGAGTCTCTGGCGGCGCATCTGGTCAAGAAGCAGCAC
AACGAGCCCCATTTCGAAGCCAAACAGGGCAGTGTGATCGCCCTGGAAAATCAGGTGCTCTATGG
CCTCACCATAGTCAACCGCCGCCGGGTACAGTTTGGCCCGCTGGAGCCGGTTAAGGCTCGGGAAAT
CTTTATTCGCTCGGCTCTGGCCGAGGGTGAGCTGCGCTGCAATGAAGCCTTCTTCAAGGCCAACCA
GGCGTTGCTCGAAGAGGTGGAAGATCTCGAACATAAGTCGCGGCGGCGCGACATTCTGGTGGATA
CAGATGCCTTGTTCGCCTTCTACGATGAGCGCATTCCACCAGGGATATACAACCAGCCCAAATTCA
ACACCTGGTGGAAACAGGCCAGGAAAGAGACCCCTGAGCTGCTCAATTTCAGCCGCGAACTCTTG
ATGCAAAGGGATGACACCCATGTATCGGCATTGGACTTCCCCGACAGCTGGCAGCAGGGCAATCT
CAGCCTCAAGCTGAGTTACCATTTTGAACCGGCGGCCGAGGATGACGGCGTCTCTGTGCTTATTCC
GGTGGCGCTGCTCAATCAGTTGGATGAAACCGATTTTGACTGGCAGGTGCCGGGGCTCAGAGAGG
AGAAGTGTGTTGCCCTGATCAAGTCCTTGCCCAAACCGCTGCGGCGCAATTTTGTGCCGGCGCCGG
ATTATGCCCGCGCCTGTGTGCAGGCGATGGAACCTTTTGCCCAGCCTTTGTTGCCCTCCCTGTGCA
AACAGCTGCTGAGAATGAGTGGCACCCGTGTCAATCCGGAGGATTTCGATCTCAACGCCATTGCC
GACCATCTGAAGATGAACTTCAAGGTGGAAGACGACAAGGGCAAACTCCTGGCTCAGAGCCGGGA
TCTCGACAGCCTCAAGGCATCATTGCAGGGGCAGGTGAACCAAGCGATACGCAAGGTTGCCGACA
AGGGCATAGAGCAAAACGCCCTGACCGAATGGAGCTTTGGCGACCTGCCGAAAGAATATCAAAGC
CGCAAAGGCAGCTATGAGGTGAAGGCGTTCCCCGCCTTGGTGGATGCCGGCGATACAGTTTCGAT
CAAATTATTTGATGACGAACAGCAGGCGCTTTCCCACCACAGACAAGGGCTGAGAAAGCTGCTGC
TTATCAATATTCCATCGCCGGTGAAACATCTACAGCAGGCCTTGCCCAACAAGGCCAAACTGGCG
ATGTATTTCAATCCGTTCGGCCAGGTGCAGATACTGATAGATGACATTATTGCCGCGGCAATTCA
GCAGCTTTTGGATGAGCAGCAGTTGGATGTCCGAAGCGCCGATGATTTTGTCAAAGCCAAGGAGA
TAGTCAGGGCCGAGCTCAATGAAACCGCAGCGGCCATCGCCCTTAAGGTGGAGCAGATTTTGACT
TTGCACCAGAAGATCCGTAAGCGTCTCAAGGGCAAAATCAGCCTGGATATCGCCTTTGCCATGAG
TGATATTCAAAGCCAATTGGACGCCCTGGTGTTCAAGGGATTTGTTGAAGCTTCCGGTTGGCGAC
GTCTTGGCGATCTGATCCGCTATCTCAAGGCGATTGAGCTGCGCCTGGACAAGCTGCCGGTGGAT
CCCAACCGTGACCGTTTGCACCTGCACAGCATTCACAACGCCGAGACAGAGTTCAAGGCCGCGCTT
GCCAAGGTGCCGCGCAGCCAGGCGGTACCGGAGGCGCTGGCCGAGGTGCGCTGGATGCTGGAAGA
GTACAGGGTCAGCTGTTTTGCCCAGAGTCTGGGCACTGCCTATCCGATATCGGAAAAGCGCATCC
TTAACCAGTTGCAGCAGTTTTAA

Occurrences/Results 9 Pentamers

The pentamer 'ATTCA' occurred 7 times.
The pentamer 'TTCAA' occurred 9 times.
The pentamer 'TCAAG' occurred 16 times.
The pentamer 'CAAGA' occurred 4 times.
The pentamer 'AAGAT' occurred 4 times.
The pentamer 'AGATG' occurred 5 times.
The pentamer 'GATGA' occurred 16 times.
The pentamer 'ATGAA' occurred 9 times.
The pentamer 'TGAAT' occurred 1 times.
The total occurrences of all specified strings is 71.
The total number of characters in the text is 3872.
First result (Total characters *9 /1024): 34.06640625
Square root of the first result: 5.836643406102517
Final result: 6.327882514012075

>Naegleria gruberi lobosa helicase (NAEGRDRAFT_58596), mRNA NCBI Reference Sequence: XM_002675000.1 helicase lobosa

mRNA Sequence

CATCAGAATCACACAAGACTATCACGCACCAAGAAATTTTTTTATTCTTGCTGCCCTCTCCTCTTT
CCTTCAGAAGGACGACACAACAAGCTATTACCACAAAGAACACCCGATTATACAAAACACATTTA
TTTGTTGAATAGTAGTACTTGTTCTCTCTTACATTTCCCACCTAAAAAGATCTTTTGATCTAATT
CCATACCCTATTTTCATCGTCATCATTGCTCCTTCTCCTTTTTAAAATTCCATAACAATTCCCTGT
AGAAATAACTTCTTGCTACAATGAATAAGAGAAAATTTGATGAAGAGGAAGTTCCTCAACAGGA
AGACCCTCTTAATTTTGTAGCGTTAGACTCCACTCTTAATCCTGCACAATTCCCAACAACAACAAT
AACAACAACAGAAAATTTAATTTCCACCAATACAAGTAACTTAACTTCCCATCCAACCGACTCTT
CCAATCCTCATCCCAACTCTGATCAACTAACACCTAAAAAGAGAAAAATTAATAGTATTTCTCCT
AGTGATTTAAATCACATTCACACATTCACTCCAGACTCTACAACATTGGATTTTAGAATTATGAA
TTTAAAATCAGCTTCCAGTTCTGCACCAGAGGATGAAGGTATTCATGATCCGAATATACCTGAAA
TAACATCTTCAGTAATGAATGACGATAGCATCTTTTTTGATAATGAATTAGATGAAAGTCTTTAC
CTTTCTTCAGCATTTTCTTCCACAGAACGTGAAGAAATAAGAATGCAAACAAAGAAATTTATTGA
AATTCAAAAATTGAATGAACAACAATATGTTCAACATGCTGTTAAATATGTAAGAAGTATTTCT
GAAGTCAACAGAATTATCAATGTTGGAGAATGGTTCGGTACAGAAGAGCTAGAAGAATCCTTTG
TTCAGGGCATTACACCCTCCCAAAGTCAAGAAAATTTCAAAATTCCACAAGTCTCCTTGATGAAT
GCAACAAATCCAAAAGCTCAAATTTCTTCAATAACCACTCCAGAAATTGTCTTTCTACCAAAGGT
ATTCTCCTCTTTGATAAAACCTCACCAGTTAGAAGGAATTAGATTTTGTTGGAGTCATATCATCC
TTCCTCCAAATGATACCCTTAGAGGATGTATCCTTGGTCACTCCAATGGCCTAGGAAAAACATTA
ACAGTTATTGCTTTTTCCTATCTCTTCCTCAAGTATAATAAGGGTAAGAAGATTTTAATCATTTG
TCCTCGTTCTGTTATTCAAAACTGGGAAAGAGAAATTTCTTCATGGTTACATTCACTAAGATTGG

AATACATTCCATGTTATGTACTTGACCATGGTAAAGACTCTAGAGTTAGTAGGTTAGAAAAACTT
CAAGAATGGGATTCAAAAGGAGGCATTCTCTTGATGTGTTTCACTTCATTTTCAAGATGGACCGA
AACACAATATTCCAACATTGATTCCAAAAGCTGTCAAAATAGATTTGATTTCGAAAAGAAGATTT
CTGACTATTTAAGAGGATGTGATCTAGCAATTGTAGATGAAGGGCATAGATTGAAGAATCCAAG
CTCATCAATCAGCAAAGCTCTTTATTGCAATATTCAAACAAGAAATCGTATTGTTTTGACAGGAG
TTCCACCTCAATTTGATCTTATGGAATACTATACCATTTTAGATTGGATTAGACCTGGCTATTGG
TCCTTGAACGAATTCAAACACTTGTTCATTGATCCTATGAATAATCACTCAATTGAATCCATCAA
ACAAAGATATTATGTACTCAAACACGAATTGAGCTATTTTGTCCACCGAAAAGATCAATCAGTAC
TTAAATCCTATCTTCCACCAAAAAGAGAATATGTTATTCAATTCCATCCTCATGATATCCAAGTA
TCACTTTATAATAACTTTATACAATCTAGAGACGGAGATGAAAAGTGTATATACTATGTTACTTG
CATGCTTCAAAAGATCCTAAACCATCCTGATTGGGTAGTTGATTATTGCCAAGCTAATAATCCAA
AAGACTCCAACACTTCATCTATCAAATTATTCTCAGATGAAAGATTGGACTTGGAGGGAAGCAAA
TCCTCACATGCTGACTTGAACTCCTATTCATTAAACACTGAGATTTCTGATGAAGATATGAGTGT
TGCTCCATCAATCAGTAACACCATGTTTAACATGCTAGAAAAAGCTGAAAAGTACAAGGATAGTT
CAGAGAATATAGAATGGGCTAGCCCTGTTCATTCCAGAAGTTACATTATGAATGCCATCGATCAT
TCTCCAAAAATGCTTTGCTTGATTAGAATGATAGAGCAATGTTTCTTATCCAATGAAAAGATCCT
AGTTTTTAGTCAATATCAAGAAACTCTAGATATTATTGAGAGAATAATTAATAATGTTAATATC
ACTACAGGTTTACCATTAGAAGACGAACATCAACCACTTAAAAGAAAGCTCAAAAGAAATTTGGA
TTATTATAGATTGGATGAATCAATGTCAGTTTCACTTGGGCAAAGTGTTGTAGACTCATTCAACT
CATCCAATGATGCACCTTTATTATTGATCAGTACAAAAGTTGGTTCCTTAGGTATTGACTTGTCT
ACTAGTCAAAAGATTGTCTTGTATGATGTTTGTTGGGATTCCTCTTGGGATAATCAAGCAGTATT
TAGATCTTTCAGATATGGTCAAACAAAACCAGTTTCCATCTATAGATTTGTCATGTATAATACTA
TTGAAAGCAAAATATTTGAAAAATTGTGTACTAAAACCTCTCCATTTAAGAATCTAAGCGAGGAT
AAAAAGGATTTCCTCAACAAGGATGACTTTGTTCCTTACACAAAACCTCCTTCCGAAAATGTAAC
CATTTCAAAAACTGACCAATTACTTTCTAATGTTTTGAACAGTGATATTAGAATTTTGAAATACC
TTCGATCAATCTTTGATTATGAAAGTCTTTATATTGAAGCTATAGATGACATGACATCAAACGAA
CAAACAAATTATAAGAGTCGGTATGAATTGGACTTTTCTAACTATACCACCAAAGATAGGAATAG
AAACAATTCTACAAGCGAGGAAGGTGACGAAAGCGAAGAAAGTGAACAGTATGAAGATGAAACA
AGTTCAGATTCAGAACAATTCGAAAGTGAAGAAGAAGAAATTGAATAAAATTTTAATCACTAGT
TTGTTCTTCTC

Occurrences/Results 9 Pentamers

The pentamer 'ATTCA' occurred 13 times.
The pentamer 'TTCAA' occurred 14 times.
The pentamer 'TCAAG' occurred 6 times.
The pentamer 'CAAGA' occurred 7 times.
The pentamer 'AAGAT' occurred 14 times.
The pentamer 'AGATG' occurred 7 times.
The pentamer 'GATGA' occurred 12 times.
The pentamer 'ATGAA' occurred 21 times.
The pentamer 'TGAAT' occurred 13 times.
The total occurrences of all specified strings is 107.

The total number of characters in the text is 3188.
First result (Total characters *9 /1024): 28.0546875
Square root of the first result: 5.296667584434576
Final result: 14.904713433782062

>ENA|GBG89619|GBG89619.1 Chara braunii helicase

mRNA Sequence

ATGCGCATAGTAGAGAGAGCTCCCAGGTCAGCGCACAGAGCGGACGGTGCCGTGGGTGGTTTCTC
CTCCAGGCGACCACCTCCTACCCCCAACTACGTCCCCTTCCGTGTCCAGAGACATGGGTTCAAACA
GCCCCTTCATTTGGTTGAAAGGGGGGGCCTTAGTCAGTGGAGAACTGTTGTAGGAGCAGGCAATT
TGTGGCGAGCCGGTCGCAGTGCTGAGGATGTGGTTGGCAGGTACTGTCAGTGGATTGCGGGACAC
ACCATACAGGGTTGCGCACTCCCTCATTGGAATTCATTTCCTGATGAGGGAAAGATAGGATCCCG
ATTTTCCCAGAAAAGGAAATATACCAGGCACGGTCCTTTGTCGCAGCGGTTGCCCGCAGAACACG
CTCGTGGAGATTCCGAGTGTAGTTTGAAAGGGAAGCCAAGGGCAAGTCTTTTGAGCGCGCTTGAT
GGTGAATGGAGAAGAACAGCGGAGGTAGAGGACGAGCAGGTGCGAGGTATACGGGATGAAGAGG
AGGAGGAAGAACTTGAAGGAGAGGAGGTAGACAGCGAAGACGATTATGAAGACGAGGAGGAGGA
AGAGGACGAGCAAGAGGAAGGAGAGGAAGAAGAGGAGGAAGAATTAGAGCGAACGCTGGAAGCG
GAAGCTTATGAAGAGGAAGGCACGTTCTTTGGCGCTGTGGAGGACGGGGAGGAAAGCAAGAGCTT
AACCATGGAAGATCGTGTGTTCAAACTCCGGGAGAGGATGCTACAGGCTGGCGATTATGGCTTAG
GGGTGAGAGAAGTACAGCGATTGTTCCCTTATCCGATTGACAAGTTTCAGAGGCTGGCCATCAAG
GCAATGTTAGAGGGTGTGTCTGTCGTGGTATCGGCGCCCACGAGCAGCGGGAAAACACTGATCGC
AGAGGCGGCAGCAGCCGTCGCCCTCGCAAGAGGGCGAAGACTTATATACACCACGCCTCTCAAAG
CCCTCTCCAATCAGAAACTGCGGGAGTTCCGTGTCAAGTTTGGGGAAGGGAATGTGGGGTTGCTC
ACCGGCGATGCCGCTGTTAACCGAGAGGCCCCTGTTTTGGTGATGACAACAGAAATCCTGAGGAA
CATGATGTACTCTAGTTTAGGTCAAATCAGCGAAGGTAGCGGGCTGATGAATGTCGACGTCATCG
TGCTCGACGAGGTGCACTACCTGAGCGACGTTTCCCGCGGAACGGTGTGGGAGGAGTCCGTCATT
TACTGTCCCAAGGAGGTTCAGCTCCTCTGTTTGTCTGCCACTGTCGCCAACTCTGATGAGCTGGCA
GACTGGATTTCCCAGGTGCATGGGCCAACTCAATTGATCACATCCACAAAACGGCCGGTACCGCT
GATGTGGCACTTTTCAACCTAAACAAGCGCTTATTCCACTTCTCAATCATCAGAGCACAGCCATGA
ACCGGCGTCTGCTTCCAAGCAGTCGGTTTGAGGAGATGCGAGAAGAAGAGCTTCCTTTTGCATTG
AGGCGGCTGGCACAGAAGAGGAGGGGCGGTCGAGAGAGAGAACGAGTCAGATCGGAAAAACAGA
GCGGCCGCCGTACTGCGGCTGTACCGCTGTCGAAGGAGGAGGAAAAACTGCTGCGGCGTACGCAG
GTTCCTCAGCTTCGTGACACCCTGCGACAACTGGCAGAGCGAAATATGTTACCTGCCATCTGGTTT
CTGTTTAGCCGAAGAGGTTGCGATCAGGCTCTCGGGATGCTTCACAGTGTGCAGCTGCTGAACGA
GAGAGAAAAATGTGCAGTGGGCGAGGCGCTCGAAAAGTTCAAGGCCACAAATGCGGAAGCTGTTC
GAGAGAGTGCCGTGAGCCCATTGATGCGCGGCCTTGCTTCGCACCATGCGGGGTGTCTTCCGGCGT
GGAAGGGGTTCATAGAGGAGCTGTTCCAGGAGGGACTAATCAAGGTGGTGTTTGCAACAGAGACA
TTGGCCGCAGGGGTCAATATGCCCGCCCGAACCACCGTGCTATCGGCGCTCAGAAAGCGTGGCGA
GAACGGACATGAGTTCCTGGCAGCCAACTCCATGCTTCAAATGGCTGGGCGCGCTGGGCGGCGCG
GAATAGATAAGCAGGGTCATGTCGTGGTTGTTCAGACACCATTTGAGGATCCAGAGGATTGCTGC

AGTCTCCTGTTCGGAGGGGTAGACCCATTGGTATCGCAGTTCACTGCCACATATGGAATGGTGCT
GAACCTTCTTCAGGGAGGAGGGCAGTATCAGCAACGCACGGAGACCGGTGGGACTCCGCTCCCAG
CGGAAAGCCAATCCCAAACACAATCCGGGCATGCGAAGATGGGGAGAACTATGGATGAGGCCAGG
GCCCTGGTAGAAAGAAGCTTTGGGAACTATCTTGGAAATGAGGCTACGGCTATGGTCAAGAAGAA
GCTGAGAAGACTCGAGCAGGAGCTCGACGACTTGGCGCTGAACCAGGAGGAGGCAGTACGCAAGT
TGAGGGAGGAGCCTCAGGATGGGAGTTTAGCGGCCTGGAAGAAGTATGTTGCACTGAAAGAGCA
AGTTCAGATGGAGAAACGGAGCTTGCGGAGGCTTCGAGACCTTCTCGACGATTTGCGAGTCGCAA
GGATGCAGCTGTTGCTTGACGACGAATTGGCGACGCAGAAATATGCTCCATTCGTAGAGCTCCGT
TTCAAAGATCAGTCCACGCGTGCTGCCAAATGCATGTGTGTGTTCTTAGTCGGGAGCATTCCCCG
ACCGTTTTTCATCACTGAAGATGAACCGTGGGAAGGAAACAAAGATTACGACGATGACGGCGATG
ACGACTTTGCAGGTCTTACTTCAAGGTGGCTGAGCACCAAAAGCAATGCTGACAATCGTGAGTTG
GGAGATCTGAACATTGGTGAAAGTGCTCATGGTGGAGGGCTTGCCCCCGGAGCTGTACGAAGCAG
CGACAGAACTGCTGGCGACGACAACCTCTTGGGAGGAGGCGATTCGTATCTGGCTGAAATTGCAG
ATGAGGGAGGGTTCGCTGCGACCCGAGGGGGCAAGGATCGTATGGGAGCTGGGGGCCCTAACCCA
GCAGATACCTATGGCGTTGACCCCTGTGGTGACGAATCGTCTGCTGGAGAAGCAAGAGGGGCCAA
CCATGCGCAGCCACAGCGGGTAGACGAAGTGCGTTATTACGTTGGGCTGGGTCCAGACAATAACT
GGTATGTGTTCACGGCCAAGTATGCAGCAAAACTTTATCGAGTCGCACCCGACGCTGTTGCTGCC
AAGTCTGCGCAACTGCGCCTGACGCAAGAGCTTCAAGAAAAGATCAGGTTAGGTTCAAGAAACTG
GGCTTGTGTGACCCCTGAGGACGACTCATCGCCTTACGGTCATGTGTGGTTGGGAGAAGGCTCGT
TCGACACGTGGCTTTGGAGTGCGTCGATTCCTCAGGCAGGGGCGCTGCCATGCAGCGTGGATGTG
TCAGATTCCGAGGTCGTGAGTGCAGAGAAAGCCCTGCAGAAACAGAGGACGGTGGTGTCAAAGCT
GAGAAAGACTCTTAAGAGCCTGCCGCAGTATAAGCAGGAGAAAAAGGCGCTGCATGCAATCCGCA
AGCAAGAGGAGAATATAACGAGCCTTGGCGGCAAGGTAGAGCGACTGAGGAAGCGTATTGATAG
GATGGCACCCACTGGTTGGAGGGAGTTTACACATGTTGTAAAGGTGCTGGAACGCCTTCGTGCAA
TCGACACAAAGGAAGAAGTGCTCTTGCCGCTTGGCCAGATGGCAGCTGCACTTCGCGGGACAAAT
GAACTATGGCTTGCCGTCGCCTTCAGTGAACCTAGCACGCACCGTCTGACGGCTGCAGAGCTGGCG
TCAGCATGTGCCACCCTTGTAACGGATATCAAAGTCCGGCAGGATTTCGGCAAAGGACTACCAAC
GCACATGACGCTGTACAGACCGTCGCAACCTGTCGTAGACTGGCTGGAAGCAATTGAACCCAAGA
GGTTGGCTTTTTGTGAAGCCCAGTTCCAAGAGGGTGTCGACATGCCAATTGAACTGGATGATCAG
TTTGCGGGCATGGTCGAGGCATGGGCGTCTGGGGTATCGTGGAGCGAGTTGATGATGGATTGTGT
AATTGACGAGGGAGACATTGCTCGCCTTCTTCGGCGGACCATAGACCTCCTCGTACAACTCCGCCA
CCTTCCGCACATCGACATCAAGGAGGAGGAGGTGGACGACGAGGGCGGCGAGGAAGGGGAGGAGG
AGGACCTGGAGGAGGAGGGCGACGAGGAAGGGGAGGAGGAGGACCTGGAGGAGGAGGGCGACGA
GGGCGACAAGGAAGGGGAGGAGGAGGAGGAGGAGGAGGAGAGCAACGAGGGCGACGAGGAAAGG
GGGGAGGAGGAGGAGGAGGAGGAGGAGGAGGAGGAGGGCGACGAGGGCGACGAGGAAGGGGAGG
AGGAGGAGGAGGATGCAGAGGAGGCAGTGAGGAGGATGAAGGGAAGGTCGAGGCCGGTGATACT
CACCGGGCGATGGCGCCAGCTCCTCCCTCACACGGCAGCACGGCAGCACGGGGCGGCACTTGAGAA
CGGTGGCAGCCCCTCCTTGAAATGGCAACGGCACTGGCAGGAATGGCCGGTGTCAGGAGGGATGA
GTGTGGCTTCGTCGTATCAGGTCGTAGGGCCACCGTTGCCGCAAACGTCAAGCTGCCATAATTTG
GCACAGCAAAGATGTCATCGTCGTTTGAAGTACGACGACGGATCCAAACCGGGGTGCAAACAATG
CTTCAAGCCTGCGGCACATGCACATGCGCCTGGTGGTGGTCACCGTCTGTGTGCAGAGTCTGCATC
CATCCATTTTGATTGGAATGGTCGTGCCAAAGTTCGCATGTGCCTCCGAAGTGCCCCTGCATCCGT
CACCACTGCAACGTTCTCGCCCACTCGCATACGGTTAAAAGAACATATCGCCGTCCCTACGAAACT

TGAGAGGACAAAAAAGAATCCAAGGCACTGCCGGTGTCAACAGTGCCGCGCGGCCCTTCACCATC
CAAGGCTGAAGGAAGCGGCCAGCTTCCTTTTTGTCCGCTGCTCGCATACCACCAACCAGGGTGAAC
GTCCGGCAGGCTTTTTGCTACCTCACACAAGAGGTAAGCAATTGGCTGTAGAAGCTCCGGCTTCG
GCAGGACAACACATGTCTGCTGCAACATCCTCATTGCACAGCAGCAGTACGTCAACAACAACAAC
AATGAAAGAAGAAGAAGAAGAGGAGAAAGACAAGGATGAAAGTACAGCATCTGTGGTTGGTGAA
ATCAGTATAGATGAGGTTACTAGTGACTGGGAGAAGTTCTCGGCCAATGTGTCGGGTGAATGGGA
TGGATTCAGCGCCGATTTCCTCGCAAATGGCGATGTATTGGAGCTGCCTGAGTCTGTTGTTCCCCA
AGCTTTTAGGGACTGGGAGGTCAGAGTCTGCGACTGGCAGACCCAATGCCCAACCCTTGCGACGA
CCGATCGCGGCATAACTTTCAAGACGATTCGGCTTCTGCCGACGGTCGGGTGCGAAGCGGACGCT
GCAACCAGGTACGACACTGTGGAGAAAAAACTGGGTGGGCAAGGTGGTGAGAGCGCTGCGCTTCT
CGCTTACAAGGATGACGGCTCTTTCATCGCTAGCTGGCATGGAGGAGGGAGGCAGAGTGAGGCTT
ATCCTGCTGACGAGAGTGGTAAGGAGCTTGCGCTTCCAATGCAGTGGACGGTCGAGCATTGTCTC
GTCGAGGGAGAGGGAGACGACGATCGATCCAGGGTTCGAATCTTGCAGTCGCTGGAGAGAACAAG
TGAGGGAGGGCTGGAATCTGTCAGAGTGAAAAAGATTACGGTTTTCCGGGAGCGATACGAGGGCC
CGTGGCGGAATGGCGAATCTTTCGGCGGATGCTCCACTGGCCTGGGAGCTTTTGCGGAAGCCCCG
AGAGCAGAGCAGTCGGCGTTAGAAGGACGGTGGAGTGGAGAAAGCTGGCGATACGATTTCAACCT
CAACCTCTCGGCACATGACAGCGGTATAGCGAACAACCTTTTCGCACTAAGAAAGGGAGAGGGAG
AGGGAGAGGGAGAGGGAGAGGGAGAGGGAGAGGGATCCTTTGAGGTTAATTGTTGTAGTGGTGC
CGCATCCACTTTGGCGAATGTGGCGGGAGATTCGTATCCGCTAAGTGGGGTTTGTGTGCGTTTGC
CGACTGGGCTGTGGTCGCGTGTGAGCATCAAGAACGAGGAAGTCAATGATCGTGAAGGAGGACGA
CTTACCGTTGAGGCGGGATGGCTCTTGGAGGAAGGAGGACGGATGATAAGGTCTGTCTGCGAGTA
TGGGCGCACCGGAGAAGTCAACAGCATGTGCATCGTCTCGGGAACACGAGCAGGAGGAGTTGGAG
CGTAA

Occurrences/Results 9 Pentamers

The pentamer 'ATTCA' occurred 2 times.
The pentamer 'TTCAA' occurred 12 times.
The pentamer 'TCAAG' occurred 13 times.
The pentamer 'CAAGA' occurred 14 times.
The pentamer 'AAGAT' occurred 9 times.
The pentamer 'AGATG' occurred 8 times.
The pentamer 'GATGA' occurred 18 times.
The pentamer 'ATGAA' occurred 10 times.
The pentamer 'TGAAT' occurred 3 times.
The total occurrences of all specified strings is 89.
The total number of characters in the text is 6371.
First result (Total characters *9 /1024): 56.0302734375
Square root of the first result: 7.485337229377177
Final result: 4.404574644026194

>ENA|EY255105|EY255105.1 UfSSH-7 SSH cDNA Library of Ulva fasciata treated with 50 micro-M CuSO4 for 6, 9, 12h Ulva fasciata cDNA clone SSH-7 similar to RNA helicase-like protein, mRNA sequence.

mRNA Sequence

AATGCAGGCCTGTGTGTTGTGCGATCAATTGGCAGGTCATAGTTGATGACCAAAGTGACCGTGCT
AACGTCGAATCCTCGAGCAATGACATCCGTGGCAATAAGGATTTTGGCATGGACAGCCCTAAAGT
CAAGGACAGCTTGGTCCCGAGCCTCCGGCTGCATATCTCCCGAAAGCTTCAAACACTTCGCGCCCA
TGCGCAGCATACTCTCATAGAGCGCCTGGGCAGTCCGTCGTGAGGCAACAAAGATGATCGTCTGC
CCAAGCCGGTCAGCATTCTTAAGGATGTACCTCGGNGNCNGACCACGCT

Occurrences/Results 9 Pentamers

The pentamer 'ATTCA' occurred 0 times.
The pentamer 'TTCAA' occurred 1 times.
The pentamer 'TCAAG' occurred 1 times.
The pentamer 'CAAGA' occurred 0 times.
The pentamer 'AAGAT' occurred 1 times.
The pentamer 'AGATG' occurred 1 times.
The pentamer 'GATGA' occurred 2 times.
The pentamer 'ATGAA' occurred 0 times.
The pentamer 'TGAAT' occurred 0 times.
The total occurrences of all specified strings is 6.
The total number of characters in the text is 306.
First result (Total characters *9 /1024): 2.724609375
Square root of the first result: 1.650639080780532
Final result: 1.9843166584006828

>Galdieria sulphuraria helicase (Gasu_22520), mRNA rhodophyta NCBI Reference Sequence: XM_005706806.1

mRNA Sequence

GAATTACTTGATGAAAAAGTTGTTATTTTTCATATGTGGAGCGCCAGGTGTGGATAAATTGTCAT
CAATAAGAATACCGCAACAAGGTTTTACACTACCCGATAATTGTGACAGCGAAGATTACCTGAAC
CGTTGTCGCTTTTCTCCGTATTATTTCTTTCTTCGACACCCAGAAGCAGGCTTAGGTCGCTTTTGT
AAACAAGTCAGTCAACACCTCTCAGAATATCAAGTCGCTATTGTAGAAGATGAACAAGGTGACAT
ACCATTATTGGAAGAAATTCACAGAGCTATAAATAGAAGGTTGAATCAAGAAGAACAAGTGGAA

GTGTTGGTTAACAAGTTTTTATTGATGGTAACAGACTTTGGTATTTGTAGAGAGGGAATTTTGA
AACAATTGGCAAGTTCAATAAGAAAGTTTGAACTGACAAAGGACGGTTTGGCTAAAACAGCCCTT
TCGAAGCAAATCTTGTGTCAGCAGCTCGCTCAACAAGTATACGCAGATTCGGTTTTCATATCTGC
CTGGGAAACGAGTGAAAATCGCGAAGCAATCATTTCCTTGACAGAGCAAAATGGATACAAAAGT
ATATCAGTGCATCAAACACAAATATGCAACCCAGTTGAGGAGATCCATAATAATGAAGAGCCAAC
TGCCTTGTTCTTATTTCTCTCAGTTTCCATTAGTAATGCCATTGCAAGTTTGAAGGAAGAACAAC
TCCACAAGTTGAGAGAATGGATTCCACGGGAAAGTATTTTGGTTGTGCTGGGCAAGTTTTACTCG
GAAACGACAACGAGTATATCAAATTTTTCAAAGTCTTTGGCTCATCGATTGAACCGAACTGTTGT
GGGCATACAGTTATGGGATGGATTATGGAAGCACTCTGATGATCTATTCAAAGCTGGATATATTC
TGATGTCATCGCTTATGTCTTGTCCATCGATTGCGCTTGTCCTTTTTGAACCAGGTCCGAATGAG
ATGATGGTAGATTCTTCTGTCATCCCTGCCGTTGATTTGGAAAGTATTATGCGAGGAAGAGAAGT
AGAAAGTGTATGGCAACTTGTATTGCGCAAATGGAATATGGAGAAAAGAAATGTTGTTTGGGAA
TGTCAAGGACTGTACAAGTTCTTGGACAATTGGTATTTAGAGCATCAAGGAAAGTGCCTTTTAGA
AATATGCGTTGTTGAAACTTGCCATTCTCTTGTAGATTCACAACGAGAGAGTCAGCAAACAGGAC
AATGCATTTCATCAGCAACTCTAAAAAACGAAGCTCTTTTGAATATCAATCCTGCTTGCTGCAAT
TTATTAGACAAGGAAAGCATTCGACATTATGCCAGTTTCCATCCCAAGTTGGTAGAAAATGCTGA
ACAGTTGTTGGAGAAGCACCGTATAGAAGTATTATTTCCATCATCCAATGAAGTGGAATGTATTA
TGGAAGCCAGTCAAACGGGAAGTTATTATCACGTTTCATTTTCTACACAAGACGATCCTCCAGCT
TTGAAGGAAACACAATGTGATTGTCCCTACTTTGCTAAACAAAAGAAAATGCAAAAGAATAGAT
TCTTATTTTGCAAGCATATTTTAGCTTTGATGATTCGGGTTCACCAGTTACTCACAGAACAACCT
ATGAATGAAGAAGTAGTTGACACTTGGAAGGAAGAAAAGAAGGAGGACGCTATGGAAACTTACC
AAGAAACGAAAGGAAATGAAACACAAGACAATACTTTGTTCACATTTTCCAATGTCGATGATGA
TGTTGACAAGAATGAGTATAGCAACGACACTCCAGAATTGTGGACAGTGAAGCCAAATGCACCTA
GAAGACTACCAGCTTTTGTGAATCATATTTCTGTTACTACAAATGCAGCACAACCCAGGAGAAAG
AAACCATTGAAAAGAAAAATTTCTCCAGTTGAAACCAGCGAATCCGAATCGGAATCCCACAGTTG
GGAAGCAAGTGGTGTGGACAATAAAACTATTCTAGTAAATGAAGCAGCTCAAGCACATAAGATG
ACAAGTCAACAAGGCGATGAGATGGAACAATCCAGTTATCCATCCAAGTCGACATCGAATGCTTT
TTACAAATATTTACTATTCGGAGATGAGTCGGATGAAGAGGAAGCATCGATTCCGCCCATTCTTT
CTCAGGAAACAAGTCTTTCAACGGAATCCTGGAATGCTTCCAAAGAGTCAAATGCGATGTTGATG
GATGGATCATCAATCTTTGGTAAAACTGAGAACACTTTAGATATTCCATCATCAAGCAAGGAAAA
TAATGCTTCCTATGAAACACAGCCTATTCAAAATGTTGATGATAGTTTGGAAGTGTTTCTTCAAA
TGGATAAAAAACCCAAGCGAAGTATTCGAGACAAACTCGCAGGTCTACAAGAATAAACTTTCATA
AAGGTTTATTGATTGCC

Occurrences/Results 9 Pentamers

The pentamer 'ATTCA' occurred 4 times.
The pentamer 'TTCAA' occurred 6 times.
The pentamer 'TCAAG' occurred 6 times.
The pentamer 'CAAGA' occurred 6 times.
The pentamer 'AAGAT' occurred 3 times.
The pentamer 'AGATG' occurred 5 times.
The pentamer 'GATGA' occurred 11 times.

The pentamer 'ATGAA' occurred 9 times.
The pentamer 'TGAAT' occurred 4 times.
The total occurrences of all specified strings is 54.
The total number of characters in the text is 2411.
First result (Total characters *9 /1024): 21.2255859375
Square root of the first result: 4.607123390739606
Final result: 7.113856366073701

>Micromonas commoda helicase partial mRNA prasinophycaea NCBI Reference Sequence: XM_002507290.1

mRNA Sequence

ATGACACTATGTAACGACAGAGGGGAATGTCCCCAAGCTGATCACAGCACATGGGCATCATTGGG
ACTGGACCGTCGAATCCTGAAGGCGTTAAAACGGAAGCAATATAACGCCCCAACAGCTGTACAGG
CTCAGGCGATTCCGCTCGTTTTGTCGGGAAAAGACGTCGTGGTTCGAGCACACACTGGGAGCGGA
AAGACGGCGGCGTACCTCTTGCCCGTCGCACACATAGTTATGCAGAATCACGCACACGGTGCCCTG
CCGAACCCTAGAGCGATCGTCCTCGTCCCTACTCGAGAGCTGGCCAATCAGGTGACGAAAGAAGC
AAGTTCAATTCTATGCCAGTGTGCCCCAACACTACGCGCAGGAGAGCTCCCAGCATCTGGATGTG
CTCCTGAAATTTTGCGGGAATTTGCCGGCGCACCTCCGGAAATACTTGTCGGCACCCCTGCTCGTG
TGGTCGAGTGTATTCGCTGTGGTTTTTTCCCTCCGGATGCTTTAAACTCTGGCTTGGACCTTCTAG
TCCTCGACGAAGCTGATATGCTTCTTTCTTTTGGCTATAACCACGATATCAAGTGCATTGCAGCT
GAAGTGCAGCGGGGATGTCAAAGCATACTTCTATCTGCGACGACAAATGAAGAGCTCACAGGAAT
GCAATCACTTGTTCTACACAATCCAGTTCAACTTGATCTAGGAAGTCTGAACAGAAACCGATGCG
GCCAAACAGAAACAAATGCGGTTCAGCCTGTTCAGTCGCCTCGAATCTCTCACTACATCATCAAA
TTACATACTGATGACAAGCTGCTGTATTGTATGGCGCTTTTCCGTCTTGGACTATGTGAAAAGAA
GACATTAGTTTTCGTTTCGCATTCGGATGCTGCCATCAGGCTTAGATTGTTCTTGGCGAAGTTCA
GCATTTCTTGTTGTGCACTACACCACGAACTTCCTTACAACTCAAGAGCCCATATTCTTCAAGAGT
ACAACCGAGGCGTCTATGATTGCATGATTGCGGTCGCCGATGACATCTCGGCGCGAACCAAAAAA
GAATCCGAGGCACGTGATCTTATGCTCGATGGGAAGCACTTGAATGCGAGTGAAGAAGTCAAAAG
GTTTGGCAGACAGAAGGATGCGCACAAAGATTTTGGGGTGGTTCGTGGAATTGACTTCAAACAGG
TGCGAACTGTGATTAATTTCGATGTTCCATCCGATGCATCTGCGTACGTGCATCAAATTGGGCGC
ACGGGACGCGGAGGAGAACATGGAAGTGCGATCACGTTTGTTTCACTATCAGAAGTGAGAAAAAT
TGAAGCCATACAACAAGATTTGAAAGAATATGGTGAAAACGGCAGTTCCCTTGTTTTCAAGCCGT
TCGGCAAACTCGCTATTGAAGATGTGGAAGCATTACGGTATCGAGCAGAGGATGTATCCCGGACA
GTAGGACGAGCTTCCGTTCGCGATGCTCGCGTAAGAGAAATTCGCACAGAACTGCTCAACTCTGA
TCGGCTTGCTGCTCACTTCGATGAAAATCCCGACGAGCTCATTTTATTGAAACATGACTTTCGCCT
TGCAAAGCAACGGGCCCTGCCTCATCTAAATCATCTGCCCAACTATCTTCGTGGGGGAAAGCCGG
TCAAGCGACTTGAGGAAAACTATACTGGTGCAGGAAATACATCTGCCCTAATTGAAGGCAAAAAA
AAAAAAATGGTGCCCAAAGTTACCCGAGCTCGGAAAAGGTACAAAACTGACAGTGCAAACAAGA
AGCTAAAGCTGAAGGATGTTTTCGCAAGTGCCAAGGGCAAATCTAGAACAGATGGTTGA

Occurrences/Results 9 Pentamers

The pentamer 'ATTCA' occurred 0 times.
The pentamer 'TTCAA' occurred 5 times.
The pentamer 'TCAAG' occurred 5 times.
The pentamer 'CAAGA' occurred 4 times.
The pentamer 'AAGAT' occurred 3 times.
The pentamer 'AGATG' occurred 2 times.
The pentamer 'GATGA' occurred 3 times.
The pentamer 'ATGAA' occurred 2 times.
The pentamer 'TGAAT' occurred 1 times.
The total occurrences of all specified strings is 25.
The total number of characters in the text is 1814.
First result (Total characters *9 /1024): 15.978515625
Square root of the first result: 3.9973135509989706
Final result: 2.256886846603624

>Raphidiophrys contractilis partial mRNA for spliceosome RNA helicase BAT1, contig 26700 centrohelida GenBank: FN392355.1

mRNA Sequence

GACGAAATTCGTCCCGTCATCAAAAAGTTCTGTCAAGAACCATACGAAATCTATGTTGACGACCA
GACCAAGTTGACTCTTCACGGTCTGCTCCAGCACCACGTTTCGGTGGAGGAGAAGGCAAAGAACC
GCAAGTTGACCGACCTGCTCGACGCCTTGGAGTTCAACCAAGTTGTCATCTTCGTCAAGAACGTC
AAGCGTGCAACTGCTTTGGATGGCTTGTTGCGCGAGTGCAACTTCCCTTCGGTGTGCATTCACTCG
AAGATGGATCAGGAGCTGAGATTGAAGCTCTACCAGGAATTCAAGGATTTCAAGCATCGTATCTT
GGTGGCCACCGACTTGTTTGGTCGTGGTATCGATATCGAGCGCGTGAACATTGTGTTCAACTACG
ACATGGCAACCGATGACGACAGCTATTTGCATAGAGTTGGTCGTGCCGGTCGTTTCGGAACAAAG
GGCTTGGCCATCTCGTTTGTGACCACCGAGGAAGACCAAAAAGTTTTGGACAACGTTCAATCTCG
CTTCGAGGTCAAGATTGTCCCTCTTCCTGACCAAATCGATGTGTCTTCGTACATGAA

Occurrences/Results 9 Pentamers

The pentamer 'ATTCA' occurred 2 times.
The pentamer 'TTCAA' occurred 5 times.
The pentamer 'TCAAG' occurred 6 times.
The pentamer 'CAAGA' occurred 3 times.
The pentamer 'AAGAT' occurred 2 times.
The pentamer 'AGATG' occurred 1 times.
The pentamer 'GATGA' occurred 1 times.
The pentamer 'ATGAA' occurred 1 times.

The pentamer 'TGAAT' occurred 0 times.
The total occurrences of all specified strings is 21.
The total number of characters in the text is 574.
First result (Total characters *9 /1024): 5.080078125
Square root of the first result: 2.2539028650321202
Final result: 7.063268840014153

>Chlorophytum comosum chloroplast, complete genome NCBI Reference Sequence: NC_053844.1: 36168-36238 tRNA-Gly

mRNA Sequence

GCGGATATGGTCGAATGGTAAAATTTCTCTTTGCCAAGGAGAAGATGCGGGTTCGATTCCCGCTA
TCCGCC

Occurrences/Results 9 Pentamers

The pentamer 'ATTCA' occurred 0 times.
The pentamer 'TTCAA' occurred 0 times.
The pentamer 'TCAAG' occurred 0 times.
The pentamer 'CAAGA' occurred 0 times.
The pentamer 'AAGAT' occurred 1 times.
The pentamer 'AGATG' occurred 1 times.
The pentamer 'GATGA' occurred 0 times.
The pentamer 'ATGAA' occurred 0 times.
The pentamer 'TGAAT' occurred 0 times.
The total occurrences of all specified strings is 2.
The total number of characters in the text is 67.
First result (Total characters *9 /1024): 0.6240234375
Square root of the first result: 0.7899515412352837
Final result: 1.74184932957827

>Monoraphidium neglectum strain SAG 48.87 unplaced genomic scaffold scaffold00424, whole genome shotgun sequence Chlorophyceae helicase NCBI Reference Sequence: NW_014013204.1

mRNA Sequence

ATGTCCTTCAACTCCTCATGCTCCGACCAGCAGCGCAAGAACGTCATGTCTGAGCTACACAGCAGC
GAGCCAGAGCTGCGGCTCCTCTACACAACGCCCGAGAGCCTGCTCAAGCCGGCGCTGCGGGAGGCG
CTGGTGGTAGGCGCCGCCCACGCATGGGGCGCATGCATGCGATGCGTGCTGCATGCCGCTTGGGTG
CATTTGACGCAGCTCGCCATGCACGGCGTGCGGGGCTGTGCATGGGACAGGTACAACAGCTGGCA
GTGTCTAGAAAAAATACGATTGAATCGCCCCTGTGTTGTGGCCGGGGGATCTCTATATGTTTATG
GAGGCTTGGAAGGTGGCTTGCGTGGTGCACGGGCTGTGCATGCACGGCCATGGCACAACGGTAGG
CCGCACATGCTGCTGAGACCTGCCAACGTGTCAGTTGACCTGCCCAGTCGGCTGCCGACAACGCCA
CTTGGCTGCCATTAGCGATGATTGGCTGCGGATAGCGAATCCCATGCTCATCCCTGACCAAACCCC
AGGAGGCTTGCGCCAGCGGCACCCTCTGCGCCTTCGCCATCGATGTGAGTGGGGCCGGCGTTGGCG
CCCATGAACTTGGACCACATCATGCCCAGCTTAGCCTCAGGGGAGCTGTATTGCTTGGTTATGAG
GCCGTGGCCACGTCATCGATCAATGCTGCCTTCACATCTCGTCTGCCGGCTGCCCTTCTCCCAATG
ACCCCCACGCCCAAACAGGAGGCTCACTGCATCAGCGAGTGGGTGAGCTGCCTGCTATCTACGCCC
TGGCCACTCGTGTACGCCCACGACCGCCTCGGGCACGTGCGCTGCGTTGCTGACGTGGTCAACTGC
GACCTGTCCTCCGTCTGTGACCCTGATCCCTGCTGCTTGCCTGCCTGCCCTTCCAACATCGGCGTG
CCATCGGTGCCGCCCGCCCTCACCCTGACCCCTGTGCAGGGCCACGACTTCAGGTGCGGTGCCTAA
AAAGCGATGATAAGCGCCCCTCCCTCTAGTTCAATGCGCAACTTGCTGGCCCGGTCTCTGGTCGCT
GGCACTCGTGGCGCCTTGTTCGCCAGACAGCGGACGCTGCTCGAGCACGGCGCCGGGGTGCGCTCT
CCCTACACCCCGTGCGCCTGCAGCCCGTGATGGATGCAGCCTACTGATCCTTCGCCCTTGACCCCT
GGACTGCGACCATCAGGCCCGCATACCTGCAGCTCGCCTCGCTCAAGGCCGACTTCCCAGGCATCC
CAATCGCCGCCATAACGGTGCGCCCGCGCCGCTCGCCCGTAGCCCAACGGCCGCACATCCTCCGCC
CCGCGCACCTGCGCCCGCCGGCAGGCGCATGGCACCGCGGGCTGCCGCAGGCGCTGCTCCTGCACC
CTGTGTGGCCCGCTACCCCATCTTTGACACTCTCACGCGTCCCCCTCTACCCACCAGGCCAGCGCCA
CGCCGCGCGTCCAAGAGTCCATCGTCCGTTGCCTCGGGCTGTCGAAGCCCCAGATCATCGCAGCCT
CGTTCAACCGCCCGAACATCGAGCTGACCGTGCGCCATAAGGCGCTGCTTGGGGCGCAGGGCGCGG
ATGACGACGTCATGGAGGTGGGATCTCATGTAGGATGATCATGTTACTATTCAACCTCACCGGGG
ATGGACACCCATGCCAACGTGTCTGCCTGCATCTGCTGTCAGGCACAGCCTCAGGGCCTGCGCTGA
GCAGCCTAACCCGTAGTGCAGCCTTCTCTTGCATCGTTGTGGTGCAACTCCCGTGATCTGCCTGAT
CATGCATGTGACTGTGGTCAAACACGCTTGCGACGTCTTCCCGCAGGACCTGCTGCGGTTCATTGG
CAACCGGCCCGGGCAGTGCGGCATAATCTACGCTCGCCTCAGGTGCGGGAAGGGGGCACATGCGG
GAGGAGGCTGCGCACCCTTGCTCTGCCGGGTGGACCTGCCAGTTGCTTCGAGCCGCTGCAAAAACT
CTCACGCTCAGGATTATACTGCCTGCCAACTTTCGTCCGTCGACATGTGGCGTCCGCATGTGGCGT
CATCAAAGCCGTCTGTGACCCCAGGAACGCCCTCATCCCGCGCATTGCTTAAACTACGCGGCAACG
CTGGCGGCATGGCTTCTTGCGCATATCTATCTGATGCAGGTCCACCTGCGACTGGCTTGCGTCGGT
CCTGTCTGGCTGTGACCTAGATGTGGCCAAGTATCATGCCGGAATGGTGAGCGCGCCCATGCCCCG

CATGTCCGCACGGCCAGCCAACCCCTCCCTCCCTTCCCGCCGCGCTCGCCTCTCTGCCGCCGCTCCG
TCACGCTGCGCGGCGTGACGGCGCGTGACGCTTGCGCCGTGCGCCGCCGGCCACGCTCGCGCTGTG
GCCCCCACCCAAAGGACCCAGACCAGCGGCGCAAGGTGCTGCGGGACTGGACCGACGGTTCGGTGG
ACATCGTGGTGGCCACGATTGGTGGGCGGGATTGATATGACATCCGATTGGCGTTTGGGGGGCGC
TGGGCACCCGCACTGCCGCTTCTGAGGGCGTGCCAGCGGCTGATGGCCGAGCTTGCAGAAAGTGG
TGCGAAGTGCTTCCATCAACCACGCCTTGCCCACCATGTCACTTCACTCACGCTACCAAGCTGTTG
CGCCTCCCCCCTTGTGACCTCCAAAATTTCAACCAGCGTTTGGCATGGGCATCGACCGGGCGGATG
TGCGCTGGGTCGTCCACTGGAACCTTCCCGGCTCAGTGGAAGGGTTCTACCAGGTCAGGGGCAGG
CAGCCACAACGTCACTTTGGTGTTTGATGAGTTGACCAGTGCGAGATCATATGCCGATCAACATG
CAGACTCATCCCTCTGCTTGCAAATGCACATCCCTGGGTAGGAGACGTGCCTTTCGCTGCCTTCCA
CACACTGTCATCCCCTTGCTGCATGCAGGAGGCTGGGCGTGCAGGGCGCGACGGCCAGCCAAGCTT
GAGCTTGGTGCGTGATCGCTTGCTTTCCAGTGCCACAGTCGGCGTGTTGATGTTGCGTGTGCTTA
CACGTTGCAGGCATGCGCACACGCACACACACACACACACACACACACACACACACACACACACAC
ACGCGCACACACACGCGCACGCGCGCGCGCGCGCACGCACACTGATTTGCACACCTTTGCCCAA
AAGGTTCATCATCTTGCGGCACCTTCCAAAACCCTCCAGGTGTATGAGTCACAAGAGGACGCAGA
AGCTGTGGCAAAGCTGGAGCGCGGAAACCGCCAGGTCGGCAGAACTGCGCTTTTCATTCACGCAA
ACCATGGTTCACTGGTCATTCCTACCCCAAAATGCTGCACAGGCATCAAGCGTGAAATGCCCAGA
GGCGTCAGGCGCCCCGTGATTTTGCCGTTGCTCAACGCCCCTGTACATTGGGCATGTGTCATGCGA
TGCTGCTCAAGCGCCCTGCGCAGGCAACAACCCTCCATCCCGGCCCGGATACCAACACCTCTGCCT
GTCCAGGGCGCTATGGCCAGCGTGGTGGACCTCATGGCCGCCCCCGGCTGCCGCCGCCGCCGCCTG
CTGGCGCACTTTGCGGAGGCGCGCGGCGCCTGCGACGCGGCCTCGGAGCTGCTGTGCGACTTCTGC
GCGGACCCGCAGGTGAGCGGGCGGGCAGGCAAGAGATGGGGCGCGCAGCGCAAGGAATTGATGCT
AACAAGGTCGCGTGCTCGTCCGCACCTGTGTCATTGTTCTGCCACATGGCACAACACAATGCTTGC
TGCCAATGGAAACCGGTTTGTCACCAATTTCTGGCCCTAGGGAGTGCGGCGGCGCCTGGACGAGC
GGGAGCGCCGCACGCACGCACGTGCGGCACGAGAGCGAGCGCGAGAGGCGCGGCGCGTGGCCGGC
GGCGAGGAAAGCGGGAGCGACGGGGTTGAGGGCGAGGAGGGTGAGGGCAGTGGCGGGGGCGATG
GCTGGGGCTCAGCGGCGCGGCTGTGCACAGCCAGTGTGCCGCCGGGAGCAGCGGTGAGGCAGCAG
CAGCAGCAGAAGCAGCAGCAGCAGCAGGAAGCTGGTGTTGTGCTGGGCGACGCTGAGGAGGAAGG
ATGCTGCGCAAGGGAGCAGCATCAGCAGCAACGCCGGCCGCAGCAGCAGCCAGACCTCGCACCCAG
GCCTGCCCTCGCGCCCCTCGGCCGCGGCCGCTACGGCAACTCAGGCGCACGGCCGGGCACGGCCTT
CAAGGCACCGACGATGGTCCAGCCGCGCGCCACGGCAGCAGCCAGTCATGCGATTCTGCCGGCAGC
CGCGGCGCCTGTATCCCCTGGCCCGGCAGTGGCCGTTGCAGTGGGCCCAAACAAGATTGGCTGGGA
GGACCCTCAGGTGGGGCTTATCGAGTCCAACACAACGCGATCCGGGCTTGTGCATGTGAGCACAG
CGTCTGGGCGGGCGCCGGTGGTGGTGCCGCGCCTGTTGAAGCGCTTCAAAGGCAGCCAGGGCTGC
GGGGTACGTCCTGGGAGCGCCGGCGGCTGCGGGGCTGCAGCGGGCGCTGAGGGCGCCACACGCAC
AGGGCCCGCGGCAGCGGCCAGGATTGGCCAGGCGGCGGTGGCAACGGCTGTTGAGGCAGCGGTAG
CGGCACATTCCAATCAGCAGCAGCAGCAGCAGCAGCAGGGTGAAGAAGGGCACAATCACAACCAA
CAGCTTGAGAACAACCAGCAGCAGCAGCAGCAGGAGCAGCAGCAGCAGCAGCAGCAGCTTCCAGC
CATGCAACAATTGCACACGCGAGTCCTGCACACGTGCATGAGCACGCACGCTGCCGCCGCGCGACG
CCCGTTCAAGCCGCCCTTGCGGCGGTGA

Occurrences/Results 9 Pentamers

The pentamer 'ATTCA' occurred 2 times.
The pentamer 'TTCAA' occurred 8 times.
The pentamer 'TCAAG' occurred 6 times.
The pentamer 'CAAGA' occurred 5 times.
The pentamer 'AAGAT' occurred 1 times.
The pentamer 'AGATG' occurred 2 times.
The pentamer 'GATGA' occurred 5 times.
The pentamer 'ATGAA' occurred 1 times.
The pentamer 'TGAAT' occurred 1 times.
The total occurrences of all specified strings is 31.
The total number of characters in the text is 4817.
First result (Total characters *9 /1024): 42.3720703125
Square root of the first result: 6.509383251314982
Final result: -1.7470273101837552
